# Supplementary material for: Haplotype-specific assembly of shattered chromosomes in esophageal adenocarcinomas
Source: Cell Genom. 2024 Jan 16;4(2):100484. doi: 10.1016/j.xgen.2023.100484 (PMC10879010; doi:10.1016/j.xgen.2023.100484)
Supplement: Document S1. Figures S1–S18 and Tables S1–S6 [file mmc1.pdf]

Cell Genomics, Volume *4*

Supplemental information

Haplotype-specific assembly of shattered chromosomes in esophageal adenocarcinomas

Jannat Ijaz, Edward Harry, Keiran Raine, Andrew Menzies, Kathryn Beal, Michael A. Quail, Sonia Zumalave, Hyunchul Jung, Tim H.H. Coorens, Andrew R.J. Lawson, Daniel Leongamornlert, Hayley E. Francies, Mathew J. Garnett, Zemin Ning, and Peter J. Campbell

Figure S1 (Related to Figure 1) - B-allele frequencies of chromothriptic region in each sample derived from Illumina X Ten sequencing. Red dots represent one allele and blue dots represent the other. Alleles are not phased so adjacent red and blue dots may be on opposite alleles. All samples show regions of loss and retention of heterozygosity.

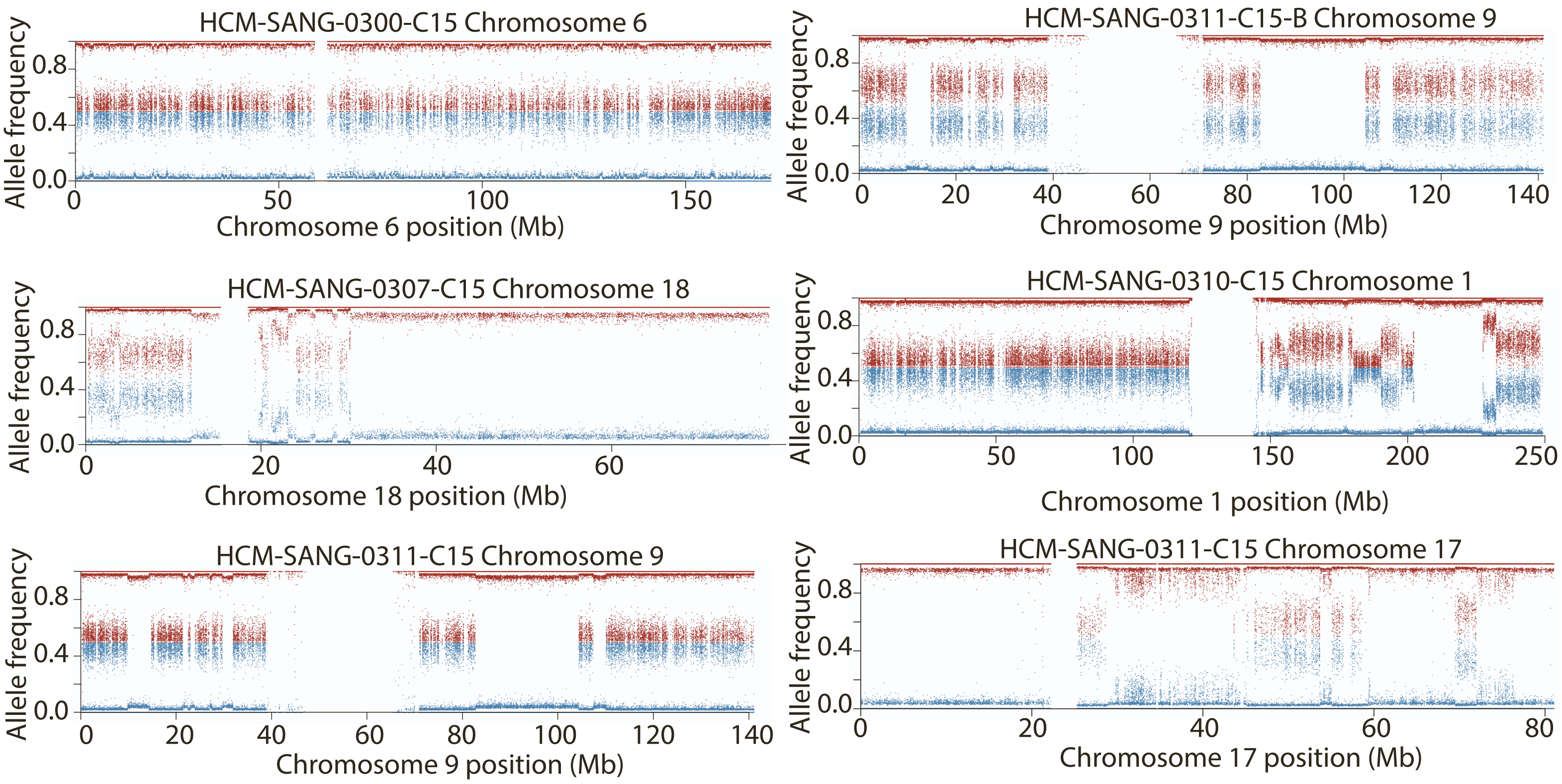

Figure S2 (relating to Figure 1) - A-E) Ciros plots containing single base substitution (SBS), indels and SVs for each sample (left). Mutational spectral for SBS (right top) and counts of indels (right middle) and SVs (right bottom) per sample. Calls were generated using Illumina short-read sequencing. HCM-SANG-0310-C15 contains 506 structural variants, many of which are retrotransposition events. F) Oncoplot showing presence of oesophageal adenocarcinoma drivers as annotated by PCAWG and OCCAMs.

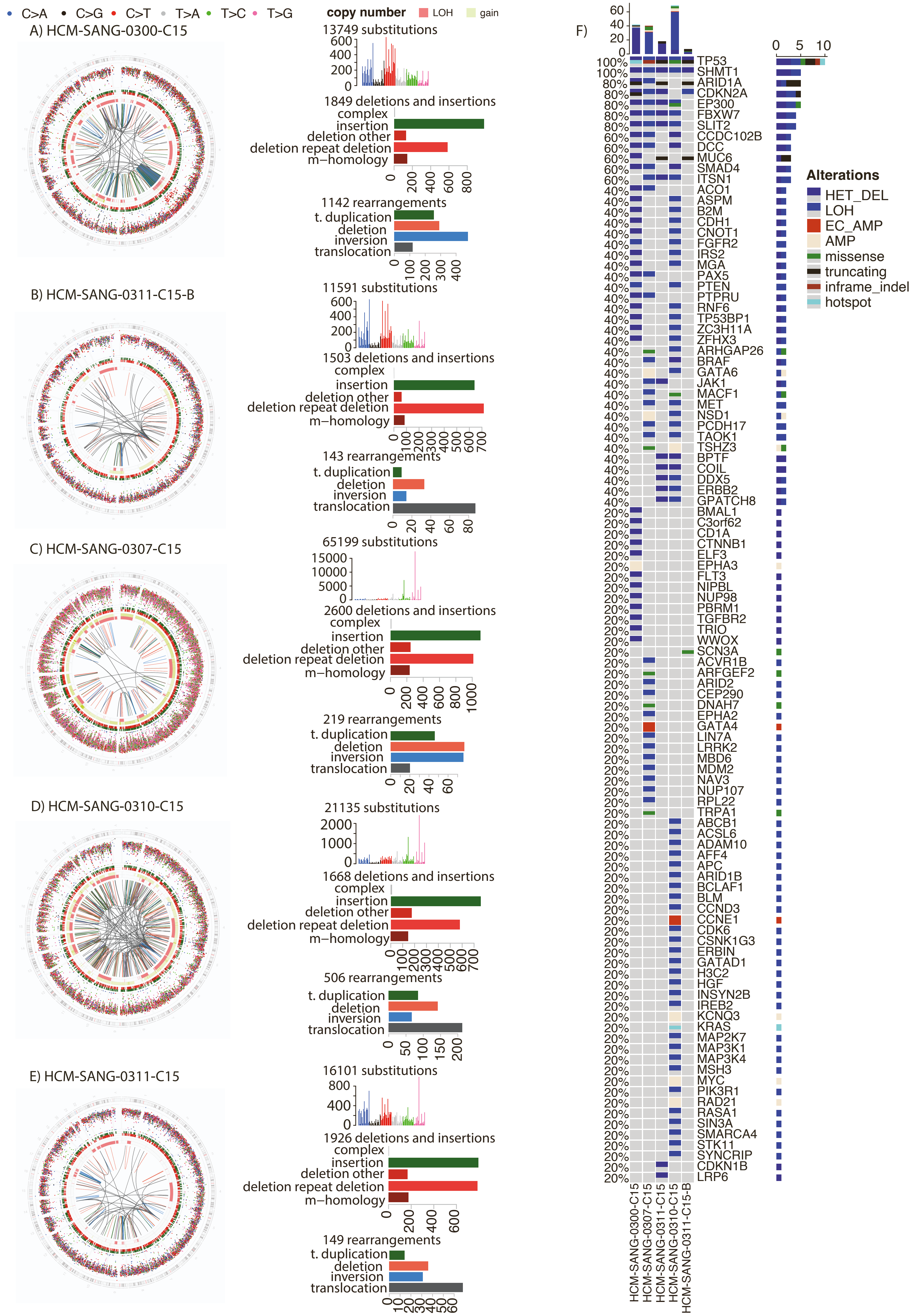
C>A C>G C>T T>A T>C T>G
copy number LOH gain
A) HCM-SANG-0300-C15
13749 substitutions
1849 deletions and insertions
complex
insertion
deletion other
deletion repeat deletion
m−homology
1142 rearrangements
t. duplication
deletion
inversion
translocation
B) HCM-SANG-0311-C15-B
11591 substitutions
1503 deletions and insertions
143 rearrangements
C) HCM-SANG-0307-C15
65199 substitutions
2600 deletions and insertions
219 rearrangements
D) HCM-SANG-0310-C15
21135 substitutions
1668 deletions and insertions
506 rearrangements
E) HCM-SANG-0311-C15
16101 substitutions
1926 deletions and insertions
149 rearrangements
F)
100% TP53
100% SHMT1
80% ARID1A
80% CDKN2A
80% EP300
80% FBXW7
80% SLIT2
60% CCDC102B
60% DCC
60% MUC6
60% SMAD4
60% ITSN1
40% ACO1
40% ASPM
40% B2M
40% CDH1
40% CNOT1
40% FGFR2
40% IRS2
40% MGA
40% PAX5
40% PTEN
40% PTPRU
40% RNF6
40% TP53BP1
40% ZC3H11A
40% ZFHX3
40% ARHGAP26
40% BRAF
40% GATA6
40% JAK1
40% MACF1
40% MET
40% NSD1
40% PCDH17
40% TAOK1
40% TSHZ3
40% BPTF
40% COIL
40% DDX5
40% ERBB2
40% GPATCH8
20% BMAL1
20% C3orf62
20% CD1A
20% CTNNB1
20% ELF3
20% EPHA3
20% FLT3
20% NIPBL
20% NUP98
20% PBRM1
20% TGFBR2
20% TRIO
20% WWOX
20% SCN3A
20% ACVR1B
20% ARFGEF2
20% ARID2
20% CEP290
20% DNAH7
20% EPHA2
20% GATA4
20% LIN7A
20% LRRK2
20% MBD6
20% MDM2
20% NAV3
20% NUP107
20% RPL22
20% TRPA1
20% ABCB1
20% ACSL6
20% ADAM10
20% AFF4
20% APC
20% ARID1B
20% BCLAF1
20% BLM
20% CCND3
20% CCNE1
20% CDK6
20% CSNK1G3
20% ERBIN
20% GATAD1
20% H3C2
20% HGF
20% INSYN2B
20% IREB2
20% KCNQ3
20% KRAS
20% MAP2K7
20% MAP3K1
20% MAP3K4
20% MSH3
20% MYC
20% PIK3R1
20% RAD21
20% RASA1
20% SIN3A
20% SMARCA4
20% STK11
20% SYNCRIP
20% CDKN1B
20% LRP6
Alterations
HET_DEL
LOH
EC_AMP
AMP
missense
truncating
inframe_indel
hotspot
HCM-SANG-0300-C15
HCM-SANG-0307-C15
HCM-SANG-0311-C15
HCM-SANG-0310-C15
HCM-SANG-0311-C15-B

Figure S3 (related to Figure 1) - Karyotyping from 20 cells from A) HCM-SANG-0300-C15 B) HCM-SANG-0307-C15 C) HCM-SANG-0310-C15 D) HCM-SANG-0311-C15-B and E) HCM-SANG-0311-C15.

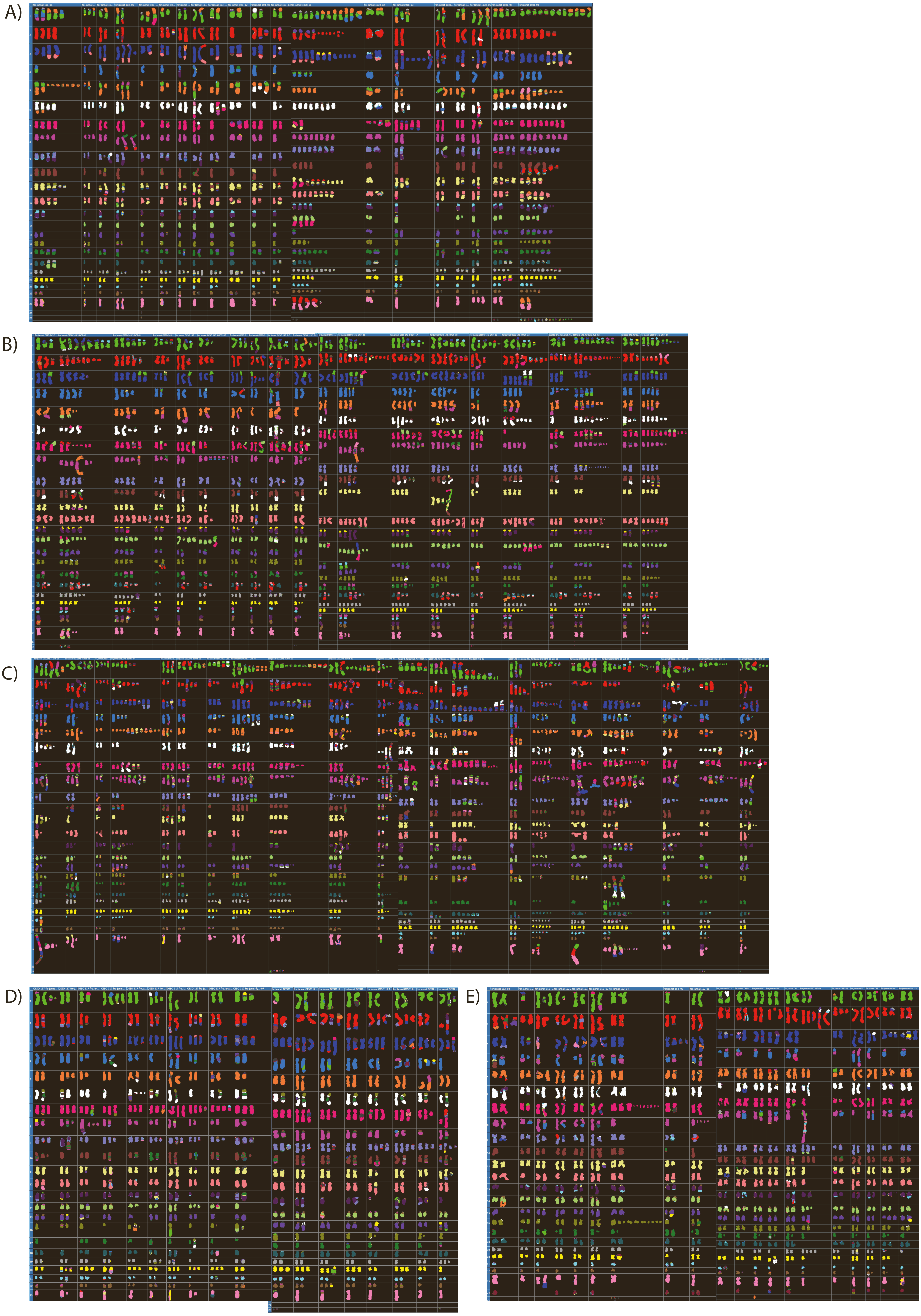

Figure S4 (related to Figure 2) - Chromosome 16 in HCM-SANG-0310-C15 assembly using hifiasm and wtdbg2. A) Rearrangement plot of chromosome 16 derived from Illumina X Ten sequencing as previously described. Notably, many copy number variants are not called by BRASS. B) Initial contigs produced for the rearranged haplotype after haplotype resolution and assembly by hifiasm. C) Final contigs produced by hifiasm after the purge duplicates step. D) Contigs produced after assembly using wtdbg2. E) Read coverage of all reads after haplotype resolution. F) Unphased B-allele frequency of chromosome 16 derived from Illumina X Ten sequencing as previously described. LOH from 0 to 15.9Mb is subclonal. LOH from 34.6 Mb to 74.4 Mb is clonal.

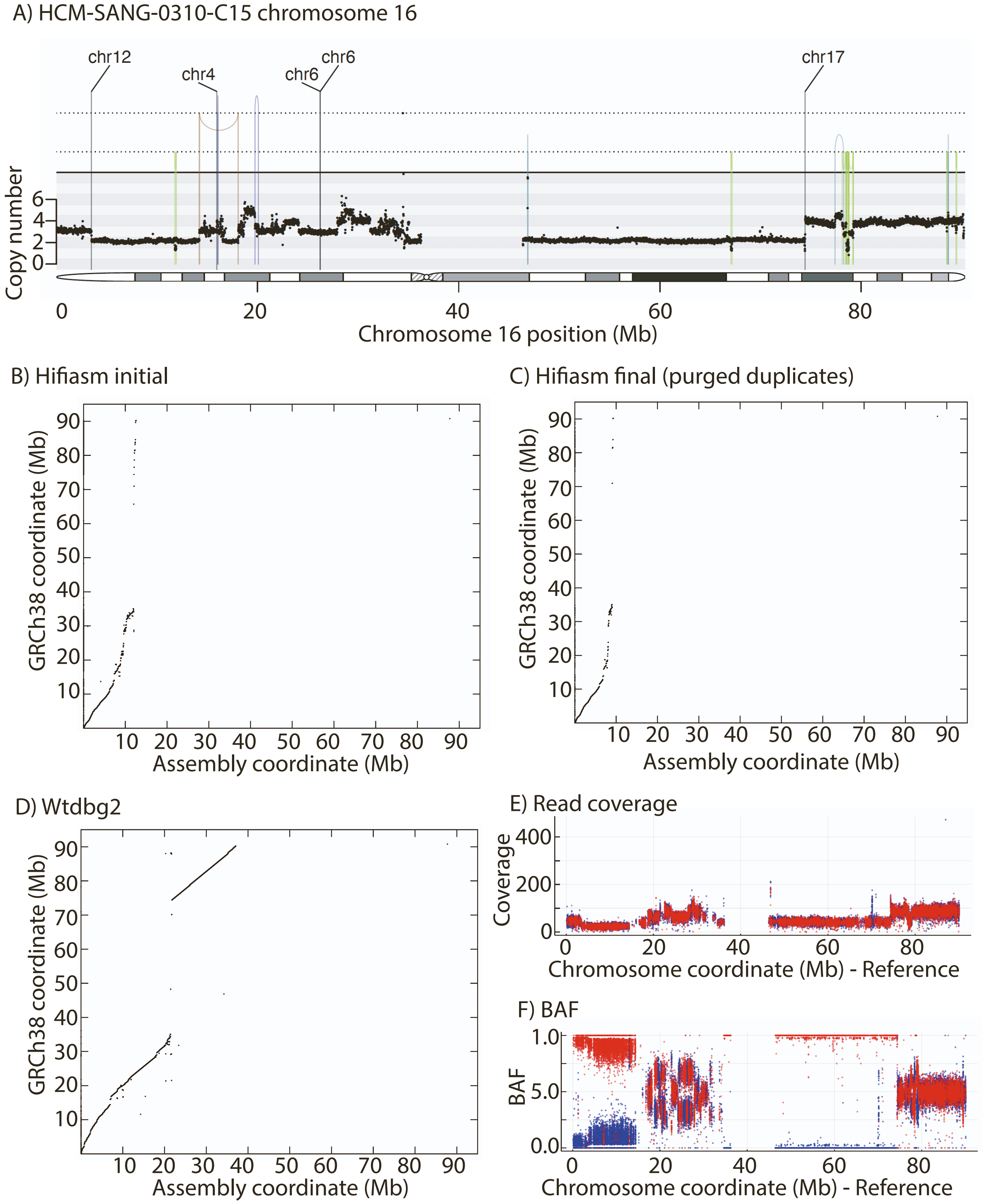

Figure S5 (related to Figure 2) - A) Assembly L90 for all samples split by chromosome. The size of the dot is scaled relative to the number of data points. Due to a highly fragmented chromosome 16 assembly with an L90 of 102, L90 is visualised on a log scale. B) Assembly N90 for all samples split by chromosome. C,F,I,L,O) Rearrangement plots as previously described. D,E,G,H,J,K,M,N,Q) Dot plots alignments of each haplotype to the reference GRCh38 genome. P) B-allele frequency of haplotype resolved short reads as previously described.

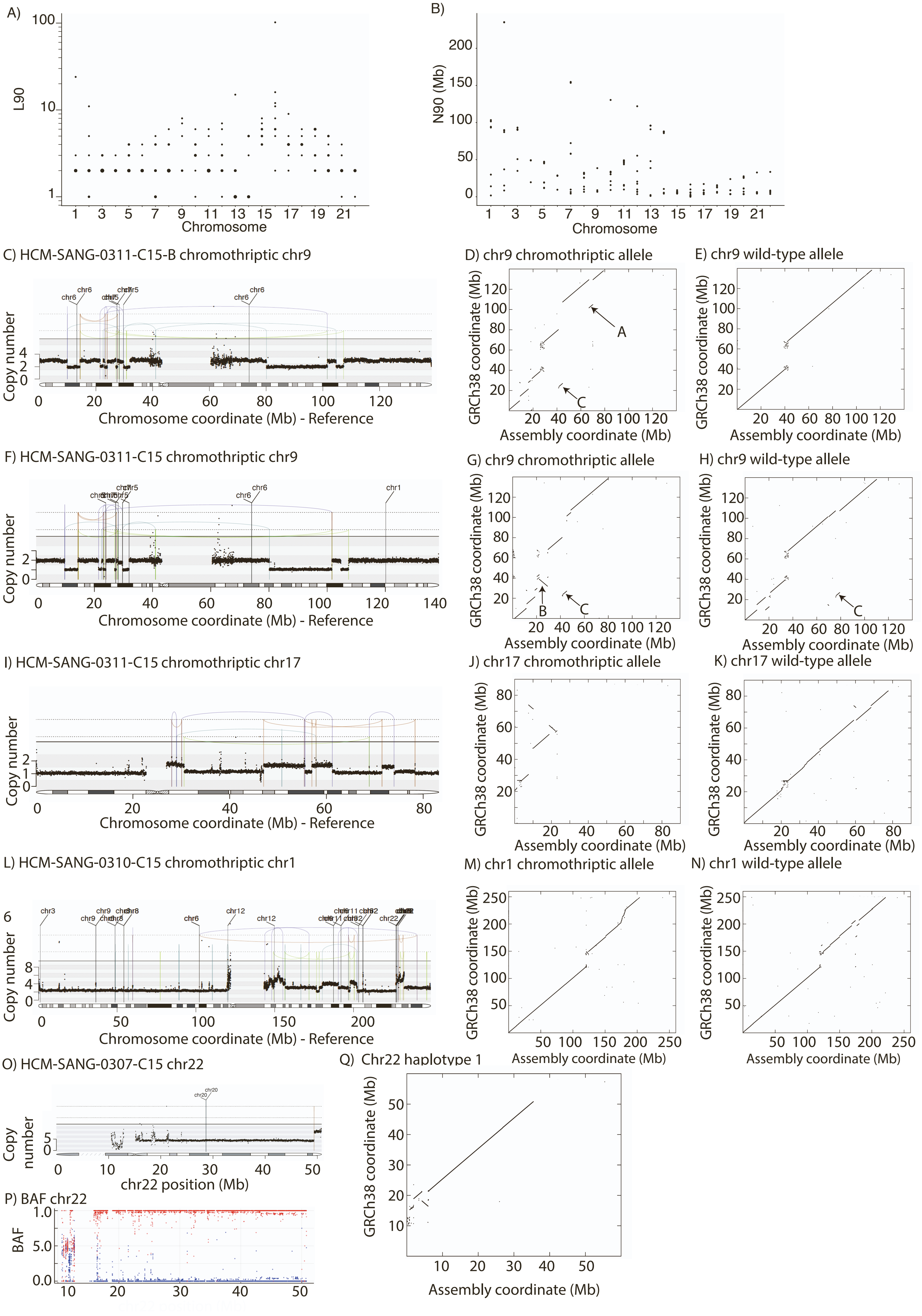
A)
L90
Chromosome
B)
N90 (Mb)
Chromosome
C) HCM-SANG-0311-C15-B chromothriptic chr9
Copy number
Chromosome coordinate (Mb) - Reference
D) chr9 chromothriptic allele
GRCh38 coordinate (Mb)
Assembly coordinate (Mb)
E) chr9 wild-type allele
GRCh38 coordinate (Mb)
Assembly coordinate (Mb)
F) HCM-SANG-0311-C15 chromothriptic chr9
Copy number
Chromosome coordinate (Mb) - Reference
G) chr9 chromothriptic allele
GRCh38 coordinate (Mb)
Assembly coordinate (Mb)
H) chr9 wild-type allele
GRCh38 coordinate (Mb)
Assembly coordinate (Mb)
I) HCM-SANG-0311-C15 chromothriptic chr17
Copy number
Chromosome coordinate (Mb) - Reference
J) chr17 chromothriptic allele
GRCh38 coordinate (Mb)
Assembly coordinate (Mb)
K) chr17 wild-type allele
GRCh38 coordinate (Mb)
Assembly coordinate (Mb)
L) HCM-SANG-0310-C15 chromothriptic chr1
Copy number
Chromosome coordinate (Mb) - Reference
M) chr1 chromothriptic allele
GRCh38 coordinate (Mb)
Assembly coordinate (Mb)
N) chr1 wild-type allele
GRCh38 coordinate (Mb)
Assembly coordinate (Mb)
O) HCM-SANG-0307-C15 chr22
Copy number
chr22 position (Mb)
P) BAF chr22
BAF
Q) Chr22 haplotype 1
GRCh38 coordinate (Mb)
Assembly coordinate (Mb)

Figure S6 (related to Figure 2) - A) Rearrangement plot of chromosomes containing a subclonal deletion as previously described. B-C) Dot plots alignments of each haplotype to the reference GRCh38 genome. D) Karyotype for HCM-SANG-0311-C15 chromosome 13. E) Rearrangement plots of chromosomes containing a subclonal duplication as previously described. F-G) Dot plots alignments of each haplotype to the reference GRCh38 genome. H) Karyotype for HCM-SANG-0311-C15 chromosome 15.

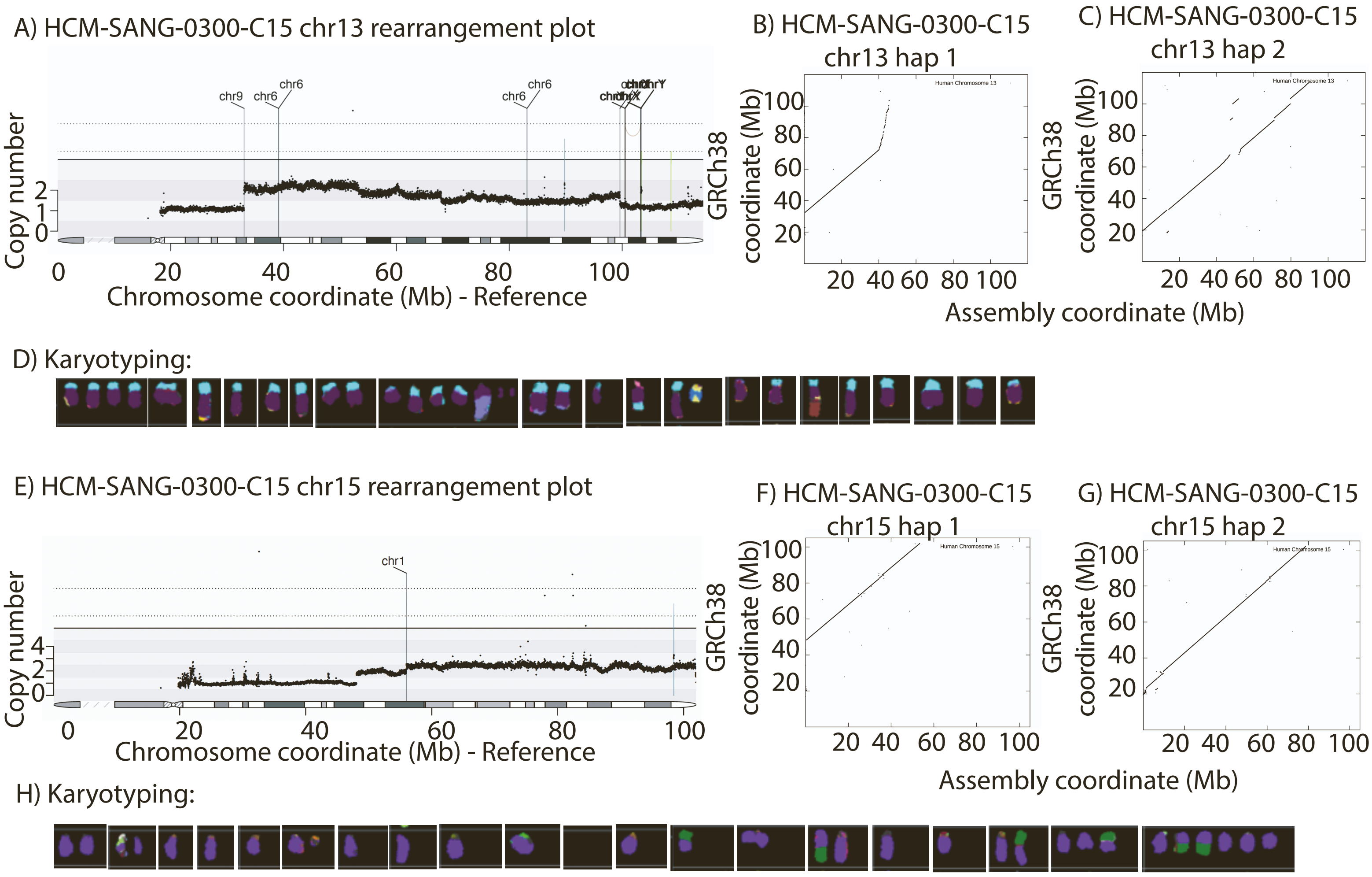

Figure S7 (related to Figure 2) - A,C,F,I,L) Rearrangement plots as previously described. B,D,E,G,H,J,K,M,N) Dot plots alignments of each haplotype to the reference GRCh38 genome.

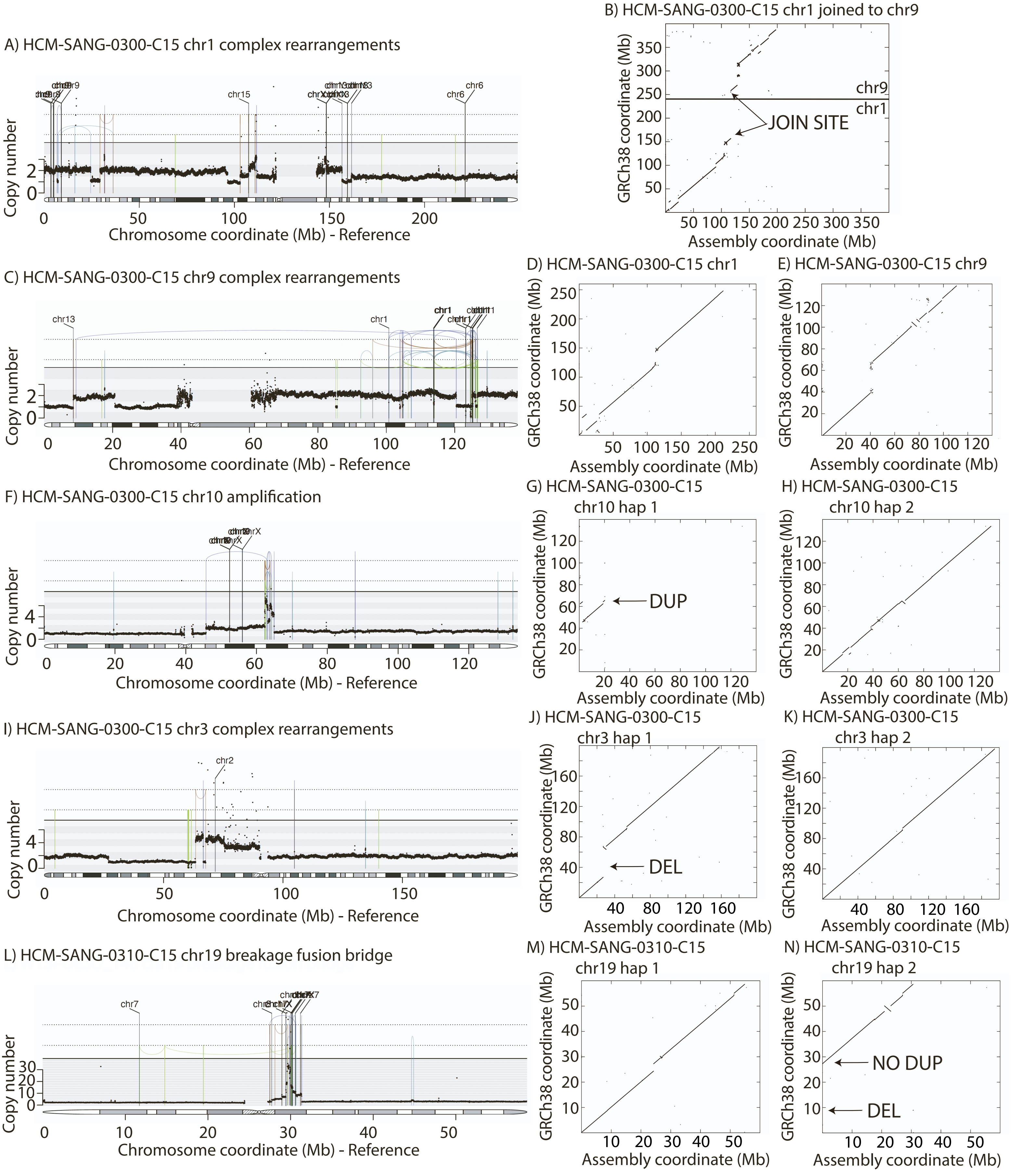

Figure S8 (related to Figure 3) - A) Number of overlapping retrotransposons when comparing calls from long-reads and short reads. B) GC content of 100 bp surrounding the retrotransposon insertion in all calls from the short-read sequencing compared to the long read sequencing specific calls. The p-value was calculated using the Wilcoxon rank-sum test. C) Fraction of retrotransposons that are in repeat versus non-repeat regions when comparing all short-read calls and long-read specific calls. The p-value was calculated using the Wilcoxon rank-sum test. D-E) Rearrangement plot as previously described for chromosome 6 in HCM-SANG-0311-C15-B and HCM-SANG-0311-C15, respectively.

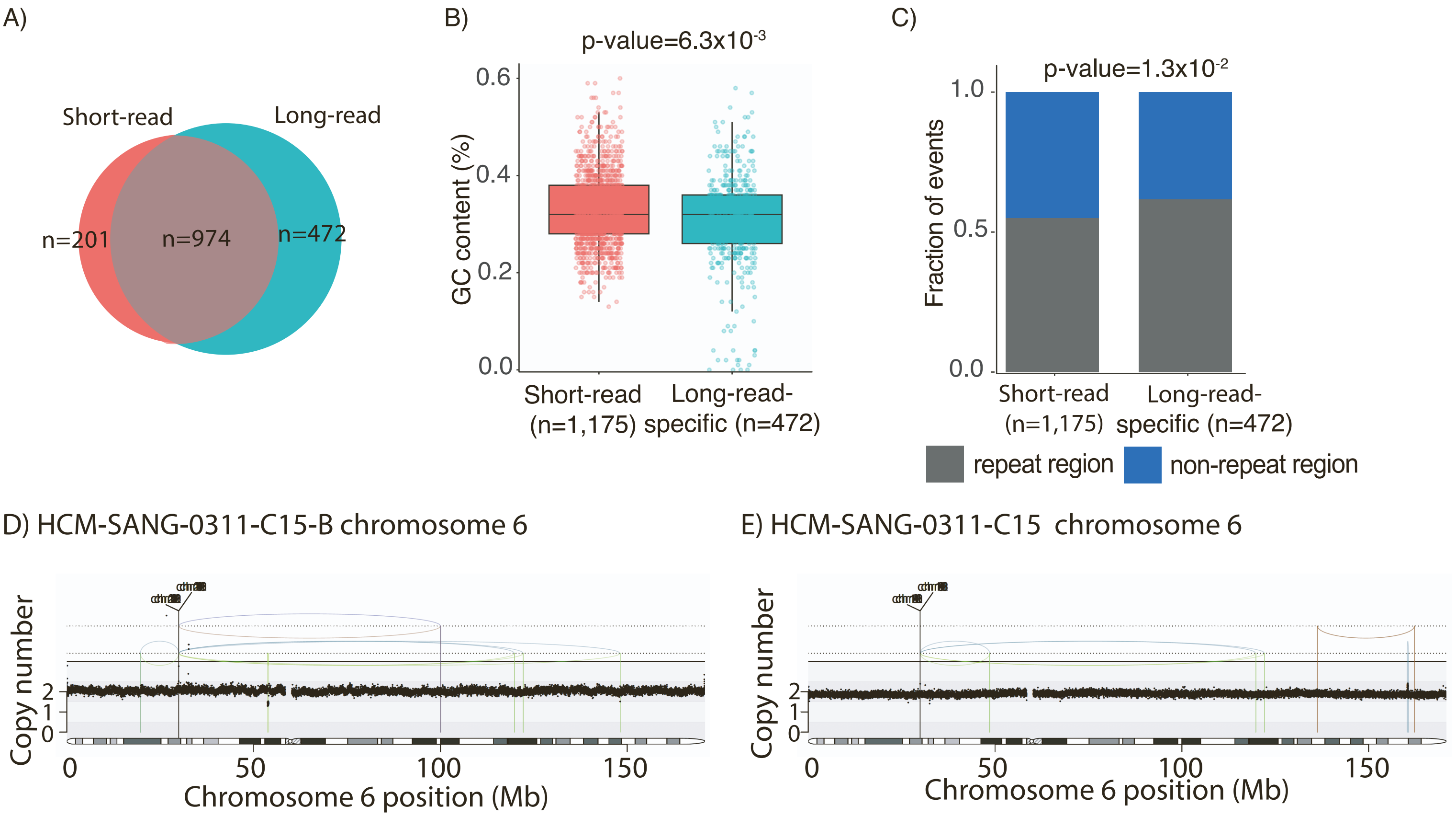

Figure S9 (related to Figure 4) - Overlap of histone modification and CTCF binding peaks on haplotypes in the initial and relapse samples. Light green: unique to HCM-SANG-0311-C15-B, blue: unique to HCM-SANG-0311-C15, dark green: shared. Most marks have a high overlap between samples.

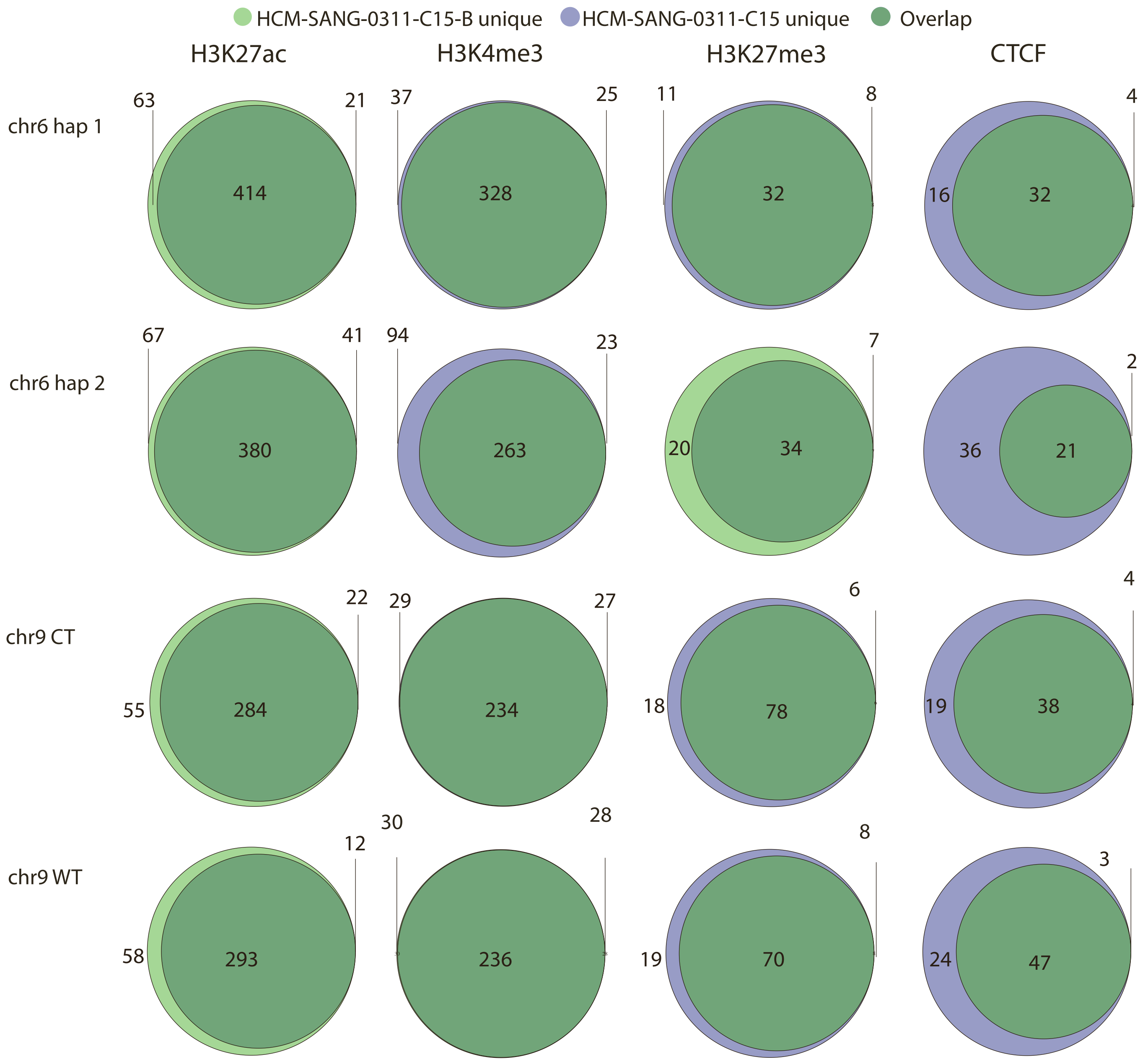

Figure S10 (related to Figure 4) - Duplication event on the chromothriptic chromosome leads to an increased number of H3K27me3 reads on the chromothriptic allele relative to the wild-type allele.

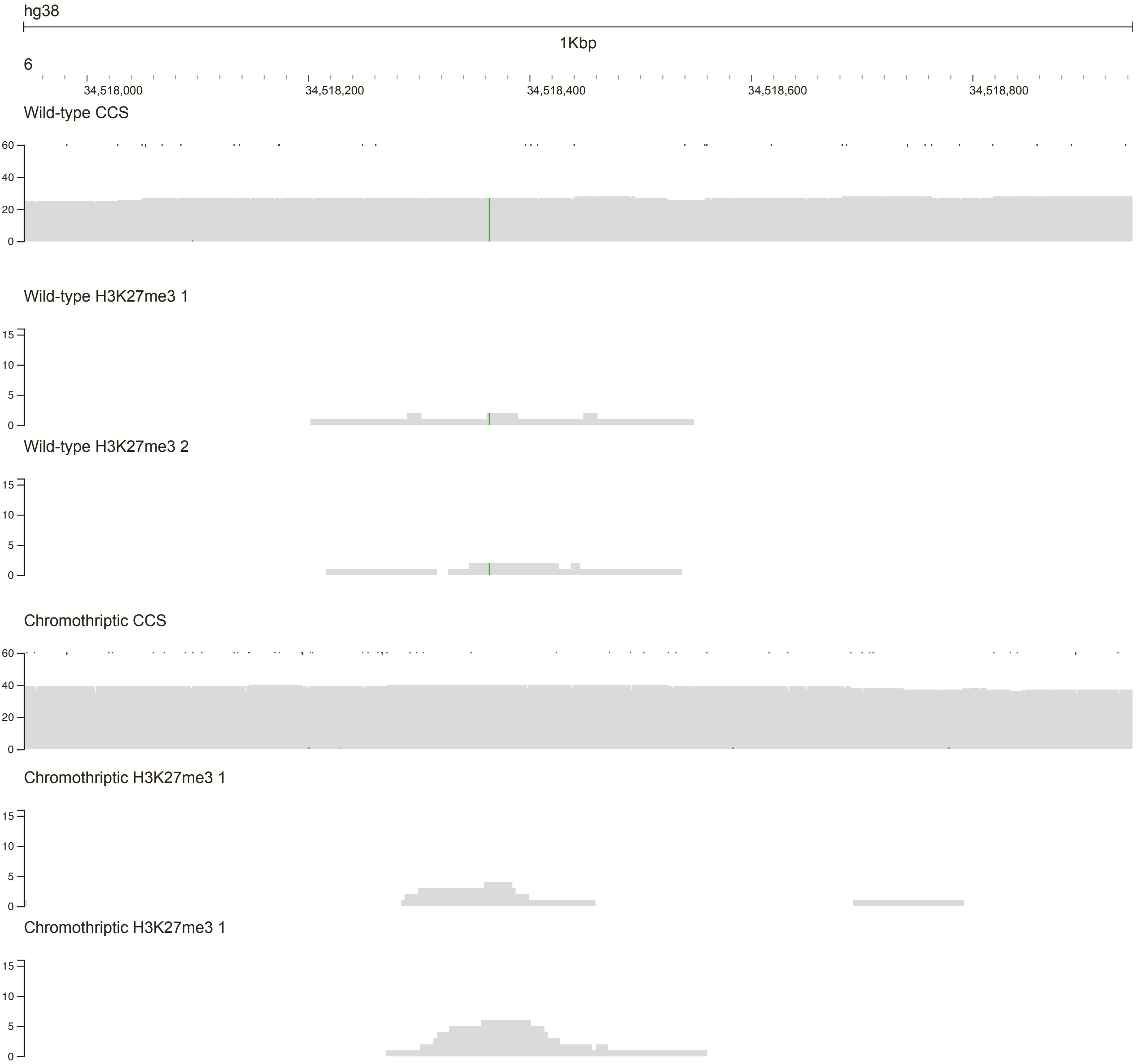

Figure S11(related to Figure 4) - Deletion event on the chromothriptic allele leads to the loss of H3K27ac and H3K4me3 peaks on the chromothriptic allele where peaks are still present on the wild-type allele.

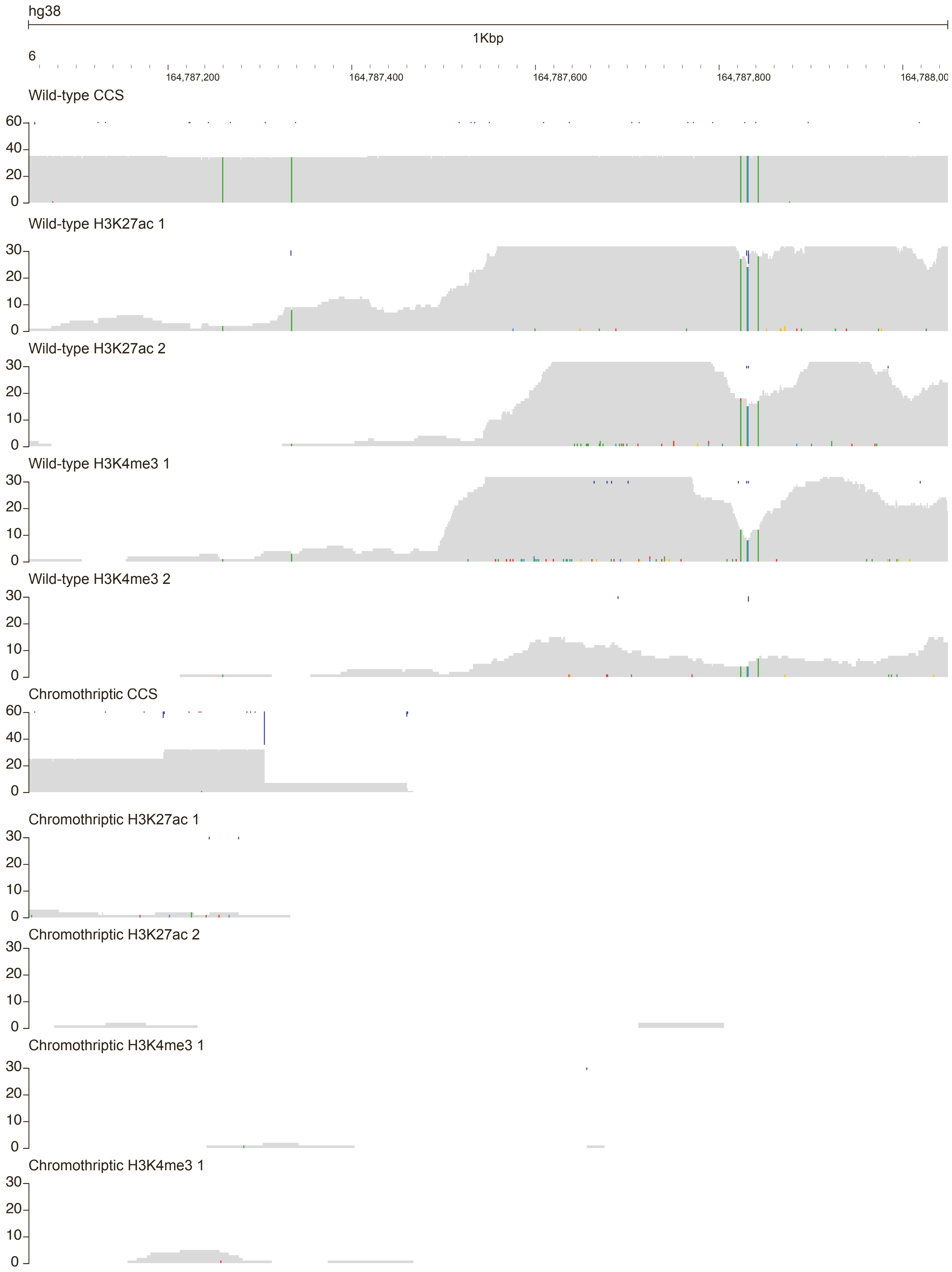

Figure S12 (related to Figure 4) - On the wild-type allele there are H3K27ac and H3K4me3 peaks, however a structural variant has occurred in the region on the chromothriptic allele leading to loss of peaks.

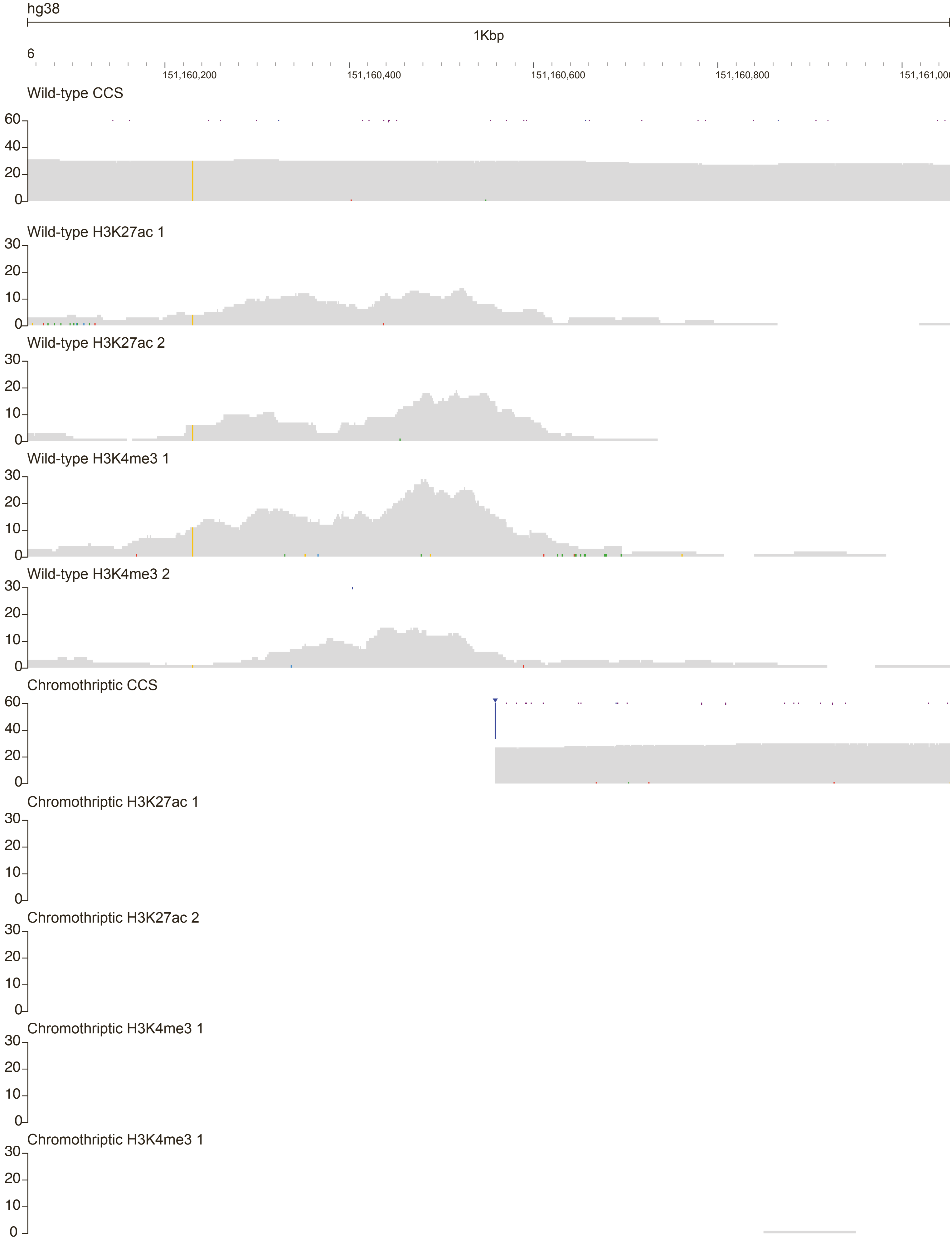

Figure S13 (related to Figure 4) - A) Distance of differential peaks with stronger binding on the chromothriptic haplotype of HCM-SANG-0311-C15-B, HCM-SANG-0307-C15, HCM-SANG-0310-C15 and HCM-SANG-0311-C15 to nearest SV relative to the peaks with equal binding on both haplotypes. B) Distance of differential peaks with weaker binding on the chromothriptic haplotype of HCM-SANG-0311-C15-B, HCM-SANG-0307-C15, HCM-SANG-0310-C15 and HCM-SANG-0311-C15 to nearest SV relative to the peaks with equal binding on both haplotypes. The p-values were calculated using the Wilcoxon rank-sum test.

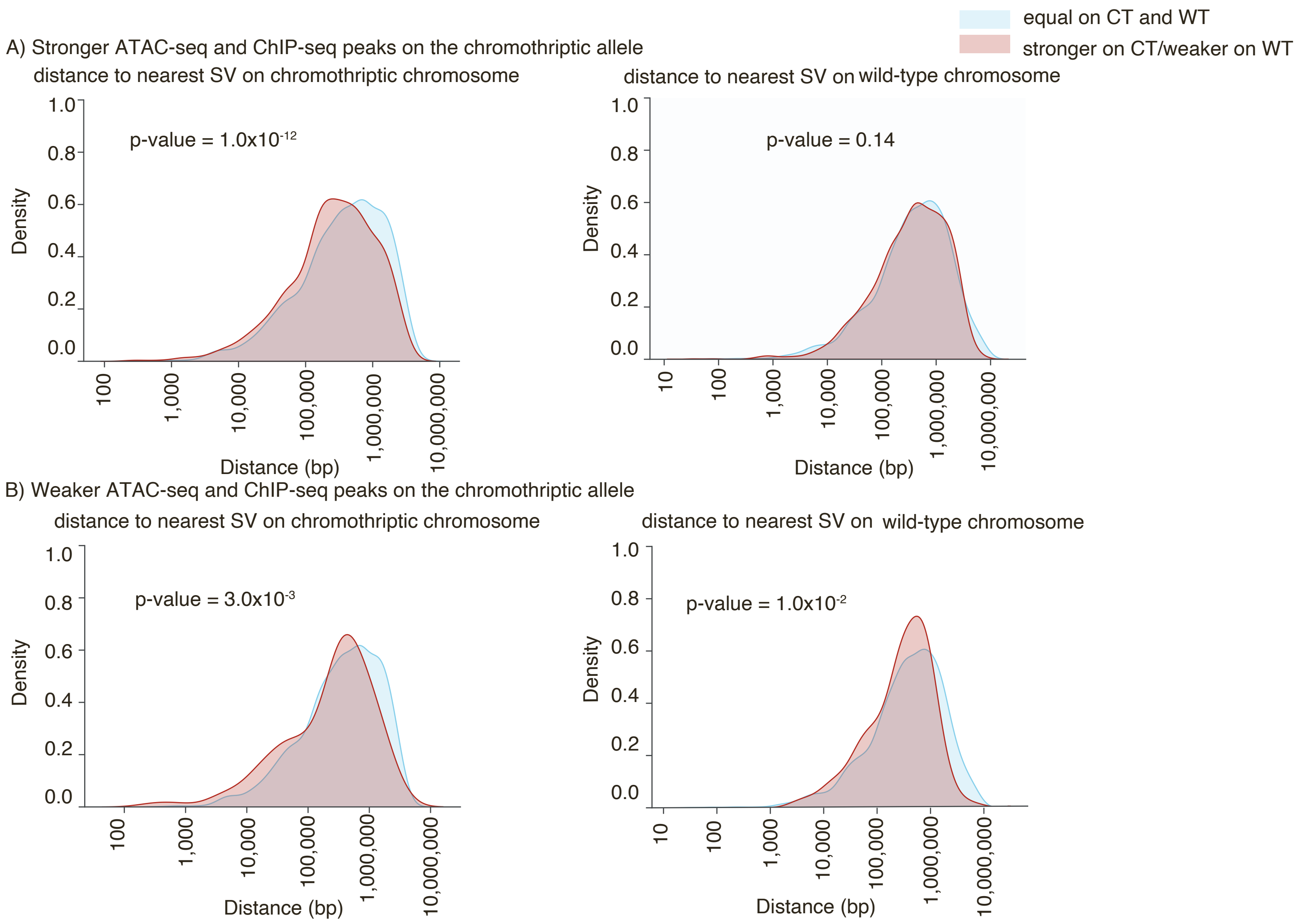

Figure S14 (related to Figure 5) - A) Distance of differential genes with lower expression on the chromothriptic haplotype to nearest SV relative to the genes with equal expression on both haplotypes. Distance on the chromothriptic chromosome (left) and wild-type chromosome (right). B) Distance of differential genes with higher expression on the chromothriptic haplotype to nearest SV relative to the genes with equal expression on both haplotypes. Distance on the chromothriptic chromosome (left) and wild-type chromosome (right). C) Distance effect on fold change of gene expression. D) Distances of genes which have higher expression on the chromothriptic allele to all differential ChIP-seq and ATAC-seq peaks (red) and distances to non-differential peaks (blue). E) Distances of genes which have lower expression on the chromothriptic allele to all differential ChIP-seq and ATAC-seq peaks (red) and distances to non-differential peaks (blue). F) Distances of non-differential genes to differential ChIP-seq and ATAC-seq peaks (red) and distances to non-differential peaks (blue). G) Distance of differential genes with lower expression on the chromothriptic haplotype of HCM-SANG-0311-C15-B, HCM-SANG-0307-C15, HCM-SANG-0310-C15 and HCM-SANG-0311-C15 to nearest SV (red), relative to the genes with equal expression on both haplotypes (blue). H) Distance of differential genes with higher expression on the chromothriptic haplotype of HCM-SANG-0311-C15-B, HCM-SANG-0307-C15, HCM-SANG-0310-C15 and HCM-SANG-0311-C15 to nearest SV (red), relative to the genes with equal expression on both haplotypes (blue). Where a p-value is stated, it was calculated using the Wilcoxon rank-sum test.

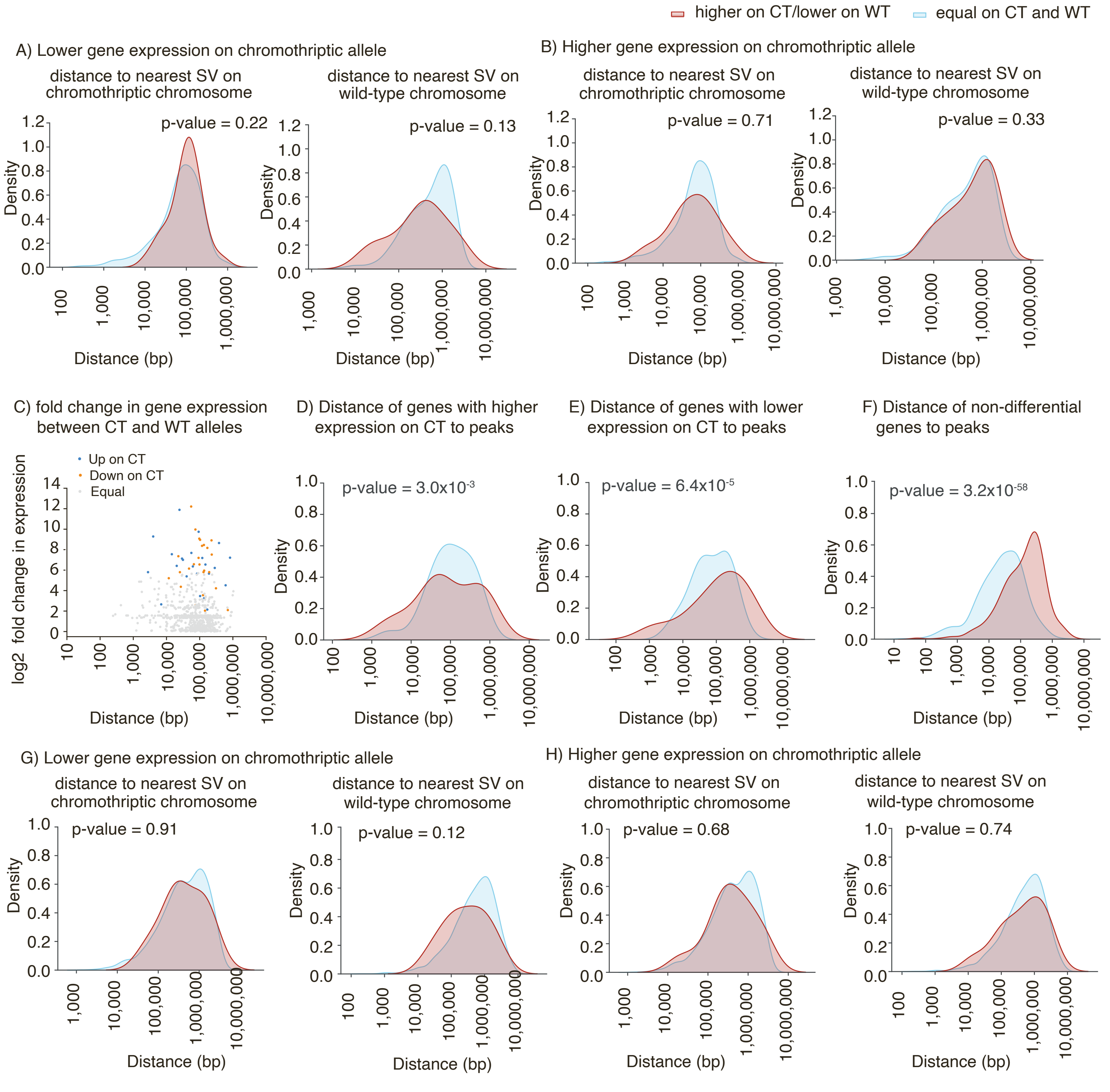

Figure S15 (related to Figure 6) - TAD sizes on wild-type versus chromothriptic chromosomes in HCM-SANG-0311-C15-B, HCM-SANG-0307-C15, HCM-SANG-0310-C15 and HCM-SANG-0311-C15 using chromosomes with evidence of chromothripsis. TAD are called using different bin sizes. TADs are inferred as regions between boundary calls. The p-values were calculated using the Wilcoxon rank-sum test.

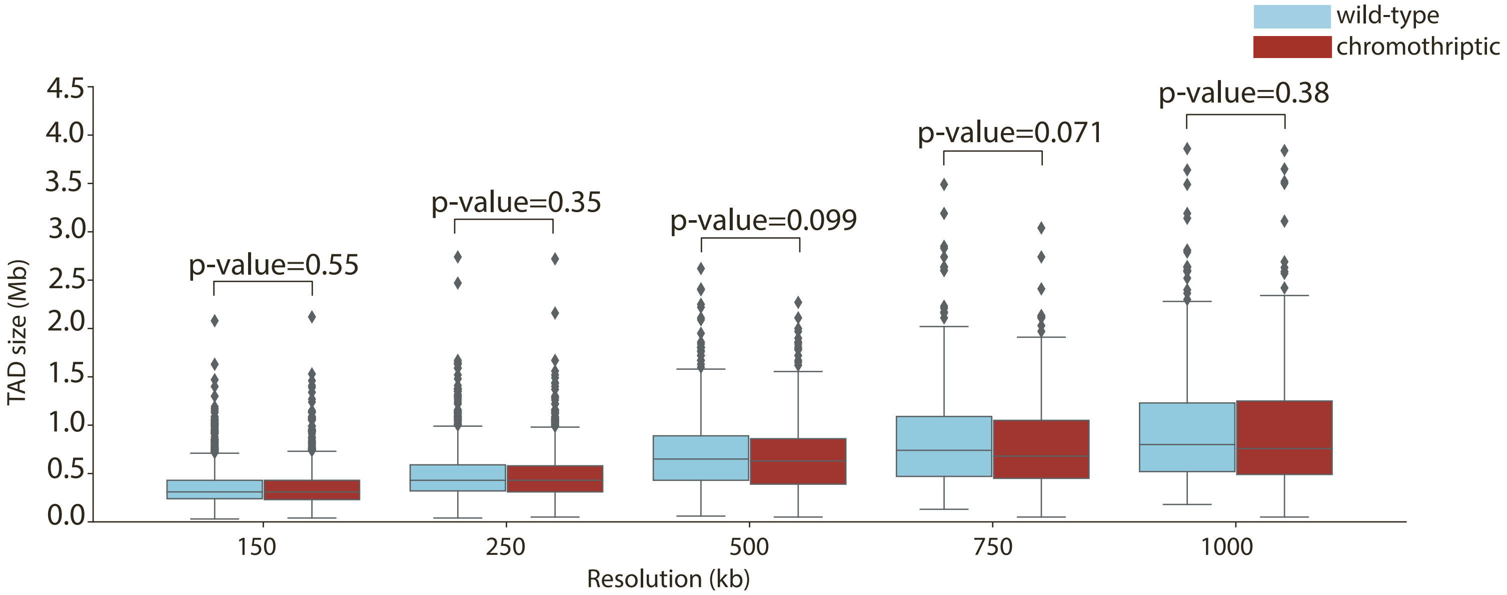

Figure S16 (related to Figure 7) - A 600kb region of the custom assemblies. Wild-type assembly is on the left and chromothriptic assembly is on the right. The regions shaded in grey are identical sequences in the two chromosomes if ignoring indels and SNPs and are equivalent to chr6:124,941,256-125,426,117 in the GRCh38 reference genome. This region contains TPD52L1 and HDDC2. Blocks in the contiguous sequences track are contiguous sequences in the reference genome. A block is contiguous but is not found in the reference GRCh38 genome adjacent to the next block. On the Iso-seq track black lines show splicing and grey boxes represent exons.

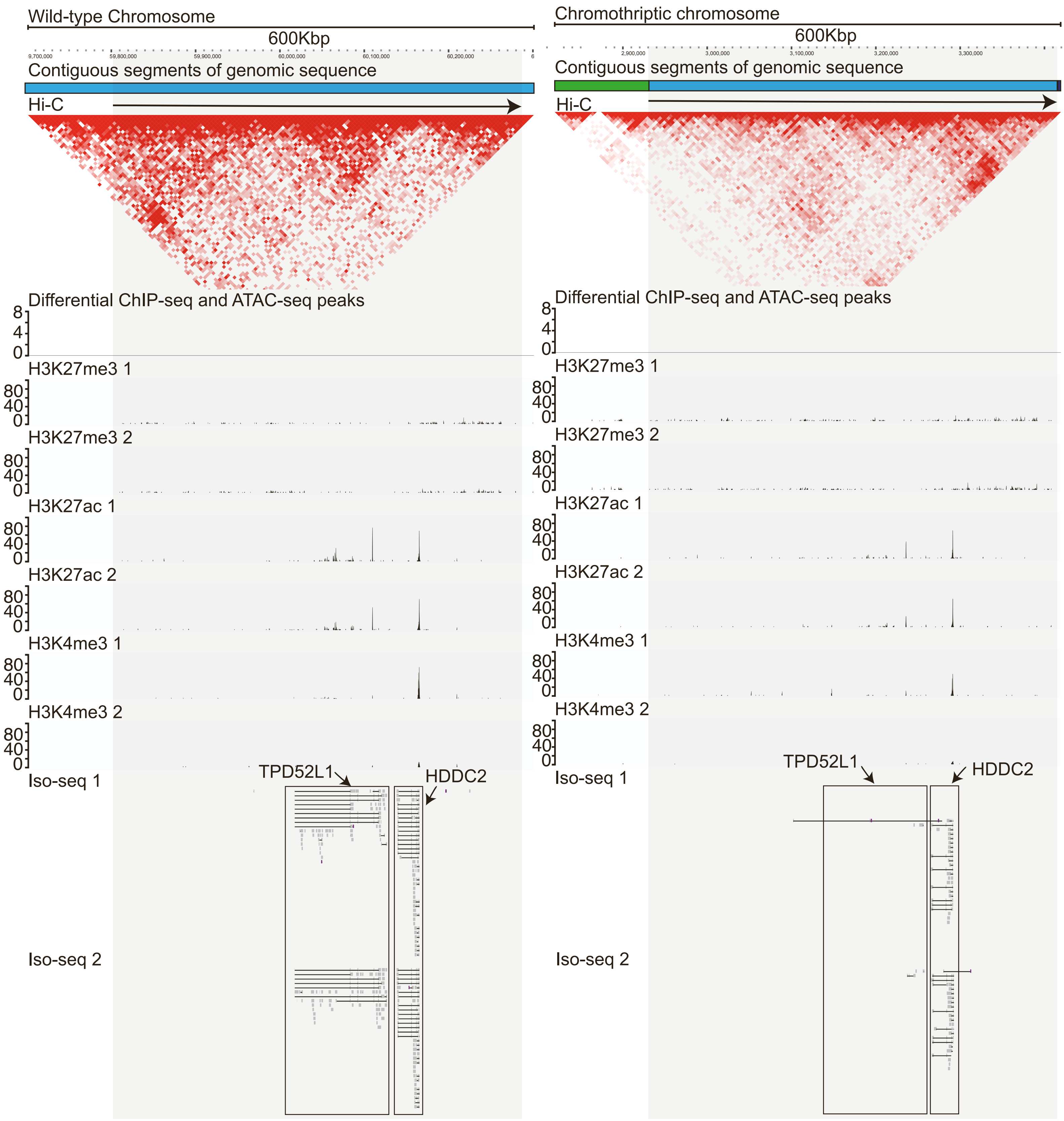

Figure S17 (related to Figure 7) - A 600kb region of the custom assemblies. Wild-type assembly is on the left and chromothriptic assembly is on the right. The regions in grey are identical sequences in the two chromosomes if ignoring indels and SNPs and are equivalent to chr6:75,536,872-75,819,360 in the GRCh38 reference genome. This region contains SENP6 and MYO6. Blocks in the contiguous sequences track are contiguous sequences found in the reference genome. A block is contiguous but is not found in the reference GRCh38 genome adjacent to the next block. On the Iso-seq track black lines show splicing and grey boxes represent exons.

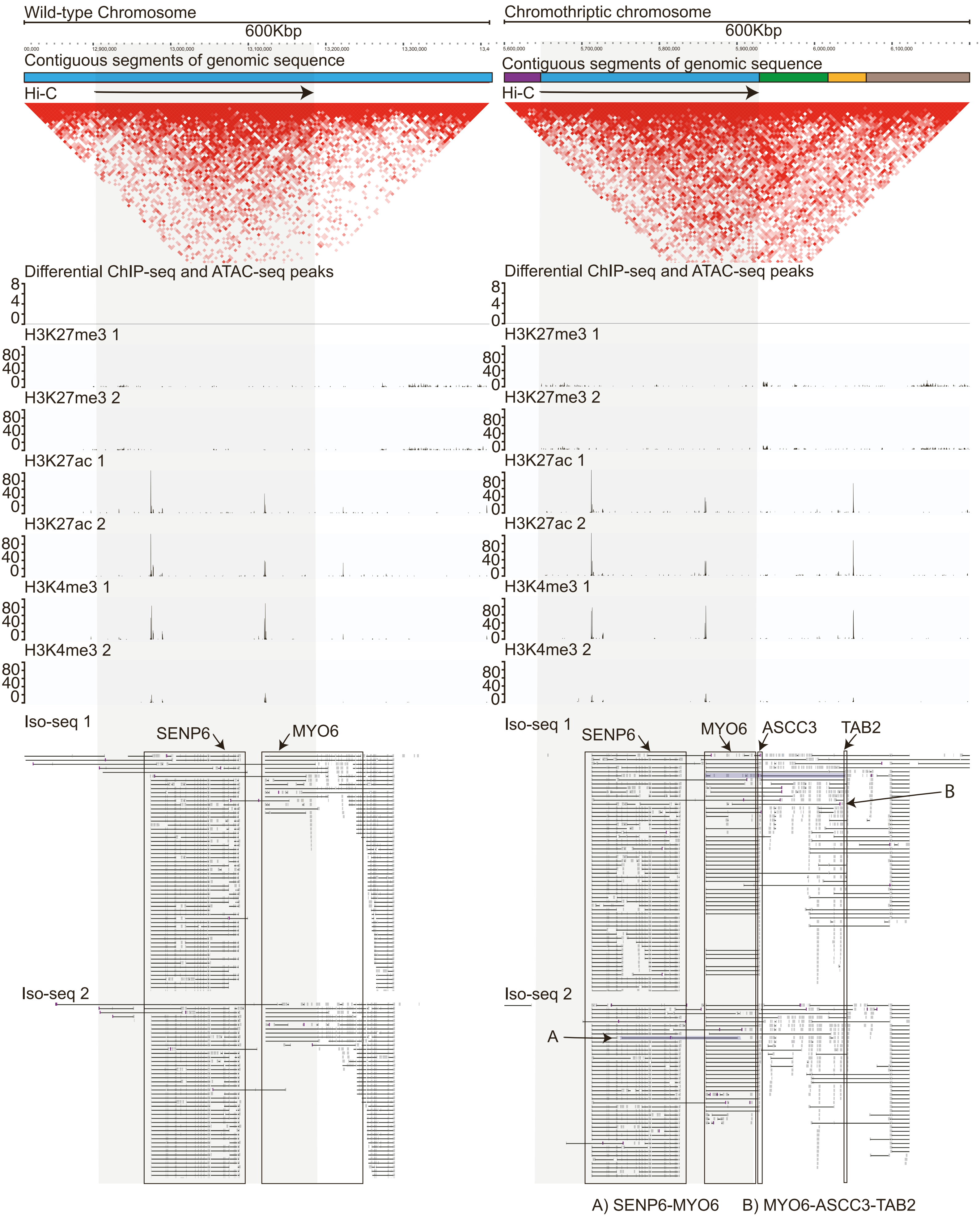

Figure S18 (related to Figure 7) - A 600kb region of the custom assemblies. Wild-type assembly is on the left and chromothriptic assembly is on the right. The regions in grey are identical sequences in the two chromosomes if ignoring indels and SNPs and are equivalent to chr6:163097370-163192248 in the GRCh38 reference genome. This region contains PACRG-AS3. Blocks in the contiguous sequences track are contiguous sequences found in the reference genome. A block is contiguous but is not found in the reference GRCh38 genome adjacent to the next block. On the Iso-seq track black lines show splicing and grey boxes represent exons.

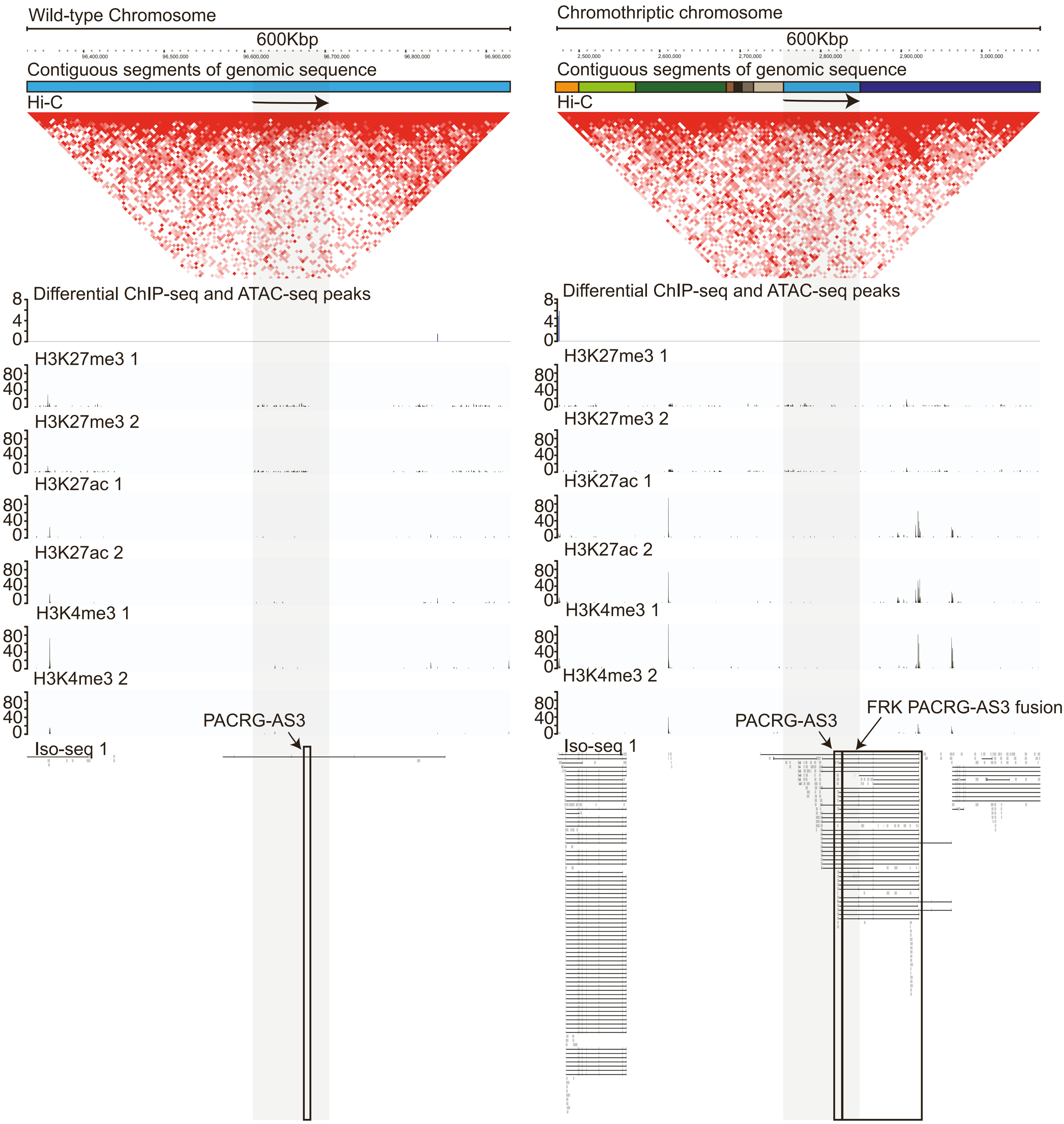

Table S1 (related to Figure 1) - sequencing statistics.

Sequencing tumour	HCM-SANG-0300-C15	HCM-SANG-0311-C15-B	HCM-SANG-0307-C15	HCM-SANG-0310-C15	HCM-SANG-0311-C15
Illumina HiSeq2500	38.6	32	40.4	42.8	41.6
PacBio CCS	45.5	72.3	71.7	71.7	54.3
PacBio CLR	41.8	68.6	101	46.4	35.6
Linked-reads	32.6	30.3	35.2	31	32.4
Hi-C	120.8	117.8	114.9	115.4	118.8
Iso-seq	7.9	12.2	8.2	7.2	6.5
ChIP-seq	8.2	8.3	6.8	9	7.3
ATAC-seq	6.4	7	6.7	5.5	6.3
Sequencing blood	HCM-SANG-0300-C15_b	HCM-SANG-0311-C15-B_b	HCM-SANG-0307-C15_b	HCM-SANG-0310-C15_b	HCM-SANG-0311-C15_b
Illumina HiSeq2500	32.6	38.1	35.9	36.9	38.1

Table S2 (related to Figure 2) - Haplotype-unaware de novo assembly statistics.

Assembly metric	*De Novo* Assembly
Sum (bp)	189,680,514
Total contigs	2,895
Average length	65,520.04
Largest contig (bp)	1,195,726
N50 (bp)	229,295
L50	239
N90 (bp)	25,358
L90	1,219
Gaps	0

Table S3 (related to Figure 2) - HCM-SANG-0300-C15 assembly statistics.

Wild-type chromosome 6 assembly (HCM-SANG-0300-C15)			
Assembly metric	CCS assembly	Scaffolds	Final assembly
Sum (bp)	148544969	148725969	148718969
Total contigs	360	54	12
Average length (bp)	412624.91	2754184.61	12393247.42
Largest contig (bp)	10457240	99230238	99925482
N50 (bp)	1475599	99230238	99925482
L50	20	1	1
N90 (bp)	204185	7746498	8732116
L90	123	5	4
Gaps	0	362	348

Chromothriptic chromosome 6 assembly (HCM-SANG-0300-C15)			
Assembly metric	CCS assembly	Scaffolds	Final assembly
Sum (bp)	122500881	122750881	122739881
Total contigs	528	176	50
Average length (bp)	232009.24	697448.19	2454797.62
Largest contig (bp)	2829437	20124411	20584246
N50 (bp)	699270	12049381	12151762
L50	53	5	5
N90 (bp)	99130	3663039	4094508
L90	229	12	11
Gaps	0	500	478

Table S4 (related to Figure 2) - Assembly statistics for derivative and control chromosomes in HCM-SANG-0311-C15-B, HCM-SANG-0307-C15, HCM-SANG-0310-C15 and HCM-SANG-0311-C15.

Chromothriptic chromosome					
Assembly metric	HCM-SANG-0311-C15-B chr9	HCM-SANG-0307-C15 chr18	HCM-SANG-0310-C15 chr1	HCM-SANG-0311-C15 chr9	HCM-SANG-0311-C15 chr17
Sum (bp)	78986462	22793105	204301382	79706155	23165814
Total contigs	12	6	66	10	7
Average length (bp)	6582205.17	3798850.83	3095475.48	7970615.5	3309402
Largest contig (bp)	37352800	11590045	69547455	25216443	13351406
N90 (bp)	3358574	10355270	13687112	5819878	2529389
L90	5	2	6	4	3
Wild-type chromosomes					
Assembly metric	HCM-SANG-0311-C15-B chr9	HCM-SANG-0307-C15 chr18	HCM-SANG-0310-C15 chr1	HCM-SANG-0311-C15 chr9	HCM-SANG-0311-C15 chr17
Sum (bp)	112838987	74359002	222558052	110163770	77837136
Total contigs	4	10	16	14	20
Average length (bp)	28209746.75	7435900.2	13909878.25	7868840.71	3891856.8
Largest contig (bp)	70332389	18680672	121190967	40816258	19671904
N90 (bp)	40086745	6884637	25618907	5427947	2563421
L90	2	6	4	5	8

Table S5 (related to STAR methods) - IDs for EGA

Cell Model Passports ID	EGA ID
HCM-SANG-0300-C15	WTSI-OESO_103
HCM-SANG-0311-C15-B	WTSI-OESO_117
HCM-SANG-0307-C15	WTSI-OESO_143
HCM-SANG-0310-C15	WTSI-OESO_148
HCM-SANG-0311-C15	WTSI-OESO_152

Supplementary Table 6 (related to STAR methods) - Clinical Data

**Clinical data**					
Tumour Tissue	HCM-SANG-0300-C15	HCM-SANG-0311-C15-B	HCM-SANG-0307-C15	HCM-SANG-0310-C15	HCM-SANG-0311-C15
Individual	1	2	3	4	2
Age	82	61	62	68	61
Cytotoxic Chemo	No	Yes	Yes	Yes	Yes
Ethnic Category	White British	Not known	Not known	White British	Not known
Gender	Male	Male	Male	Male	Male
Lines Therapy	0	0	2	2	2
Primary tumour	Yes	Yes	Yes	Yes	Yes
M Pathology	M0	M0	M0	M0	M0
N Pathology	N3	N2	N0	N0	N2
T Pathology	T3	T3	T3	T2	T3
Prior Drug	NA	Unknown	Unknown	Capecitabine	Capecitabine
Prior Drug	NA	Unknown	Unknown	Cisplatin	Dexamethasone
Prior Drug	NA	Unknown	Unknown	Epirubicin	NA
Therapy Outcome	NA	Unknown	Progressive disease	Partial Response	Stable disease
Sample collection	Surgical	Laproscopy	Surgical	Surgical	Surgical
Smoking Status	Unknown	Non-smoker	Non-smoker	Ex smoker	Non-smoker
TNM Stage	IIIC	IIIB	IIA	IB	IIIB
